# The Marine Side of a Terrestrial Carnivore: Intra-Population Variation in Use of Allochthonous Resources by Arctic Foxes

**DOI:** 10.1371/journal.pone.0042427

**Published:** 2012-08-03

**Authors:** Arnaud Tarroux, Joël Bêty, Gilles Gauthier, Dominique Berteaux

**Affiliations:** 1 Chaire de Recherche du Canada en Conservation des Écosystèmes Nordiques and Centre d’Études Nordiques, Université du Québec à Rimouski, Rimouski, Québec, Canada; 2 Département de Biologie et Centre d’Études Nordiques, Université Laval, Québec, Québec, Canada; Institut Pluridisciplinaire Hubert Curien, France

## Abstract

Inter-individual variation in diet within generalist animal populations is thought to be a widespread phenomenon but its potential causes are poorly known. Inter-individual variation can be amplified by the availability and use of allochthonous resources, i.e., resources coming from spatially distinct ecosystems. Using a wild population of arctic fox as a study model, we tested hypotheses that could explain variation in both population and individual isotopic niches, used here as proxy for the trophic niche. The arctic fox is an opportunistic forager, dwelling in terrestrial and marine environments characterized by strong spatial (arctic-nesting birds) and temporal (cyclic lemmings) fluctuations in resource abundance. First, we tested the hypothesis that generalist foraging habits, in association with temporal variation in prey accessibility, should induce temporal changes in isotopic niche width and diet. Second, we investigated whether within-population variation in the isotopic niche could be explained by individual characteristics (sex and breeding status) and environmental factors (spatiotemporal variation in prey availability). We addressed these questions using isotopic analysis and Bayesian mixing models in conjunction with linear mixed-effects models. We found that: *i*) arctic fox populations can simultaneously undergo short-term (i.e., within a few months) reduction in both isotopic niche width and inter-individual variability in isotopic ratios, *ii*) individual isotopic ratios were higher and more representative of a marine-based diet for non-breeding than breeding foxes early in spring, and *iii*) lemming population cycles did not appear to directly influence the diet of individual foxes after taking their breeding status into account. However, lemming abundance was correlated to proportion of breeding foxes, and could thus indirectly affect the diet at the population scale.

## Introduction

Individual variation in foraging behaviour can reduce competition for resources among individuals, with benefits translating into long-term stability at the population level [1], [2], [3]. Studying individual variation is a critical aspect of population ecology because it accounts for individual heterogeneity, in particular when it comes to resource use [4]. Therefore, it is not surprising that questions linked to niche variation within populations have stimulated ecologists for decades, whether in relation to resource use [3], [5] or other ecological factors [6], [7]. Understanding variation in resource use and trophic niche yields insights into community structure [8] and predator-prey dynamics [9]. It is acknowledged that intra-population variation in resource use can be related to spatiotemporal variation in resource abundance [10], [11]. However, causes of variation within populations remain poorly known [2]; when all individuals have access to the same types of resources, why do some specialize? Proximate factors, such as competition, have been proposed [12], but it is still unclear which factors can ultimately trigger the use, or not, of a different resource by a given individual.

Individual variation in resource use is common and has been described in a wide range of taxa [1], [13]. For instance, the degree of individual variation can be explained by factors such as inherited foraging behaviour [1], habitat partitioning [12], or the occurrence of geographically distinct sub-populations [10], [14], [15]. Extreme cases of individual variation, such as long-term dietary specialization, can also occur in sympatric populations [1], [16]. Alternatively, individual variation may be dynamic and change rapidly over time within a population [11]. Matich *et al.* [17] recently suggested that future studies should address these questions, especially for apex predators because they both affect and are affected by changes in prey populations. Moreover, the use of allochthonous resources, i.e., resources originating from distinct ecosystems, can have far reaching implications on intra-population variation in resource use [18]. Input of allochthonous resources, whether through active (e.g. moving consumers or prey) or passive transportation [18], can indeed alter trophic dynamics at several levels (i.e., from top predators to plants) in receiving ecosystems [4], [19]. Temporal variation in the trophic niche of top predators facilitates long-term stability of predator populations in variable environments [14], [20]. When these predators forage in more than one food web, it may also link otherwise discrete food chains across ecosystems [17], [18], thereby potentially affecting the flow of resources between ecosystems [19]. Identifying the factors generating intra-population variation in top predators is thus also crucial to better understand how food webs are structured and connected to each other.

Here, we investigated the effects of spatiotemporal variation in resource abundance and individual characteristics on the variation in individual resource use within the population of a key arctic terrestrial predator, the arctic fox (*Vulpes lagopus*, Linnaeus 1758). This species is an ideal study model for such questions; considered as an opportunistic specialist because it preys preferentially on small rodents, it can also take advantage of other prey like marine mammals and birds when available, hence sometimes behaving as a generalist [21]. Moreover, arctic foxes are extremely mobile and can use the sea ice extensively in winter and spring, thereby using both terrestrial and marine food webs [15], [22], [23], [24]. We examined individual patterns of resource use in a small population in the Canadian High-Arctic during six years from spring to summer, when breeding foxes rear pups at dens. Although this is not a longitudinal study, because only few individuals were recaptured within or across years, the random sampling of animals allowed us to interpret individual variation in resource use at a fine temporal scale.

When sequential sampling is not possible, stable isotope analysis can provide a global picture of the diet integrated over a specified time span, which depends both on the consumer species and tissue considered [25]. The use of stable isotope techniques to address questions about population trophic niche width and among-individual variation in diet is growing [26], [27], [28], along with new tools proposed to measure and compare among- or within-population differences [29], [30], [31], [32]. However, linking isotopic and trophic niches is not always straightforward [33]. For instance, isotopic niche width depends not only on the number of prey types consumed but also on the variation among their isotopic ratios [34]. Two populations with the same trophic niche can therefore have different isotopic niches if prey isotopic ratios differ. It is thus essential to ascertain that potential food sources are the same for all groups compared and are well differentiated in the isotopic space. The natural system that we used was characterized by a strong marine-terrestrial isotopic gradient and a limited number of isotopically-distinct food sources. Therefore, we assumed that the isotopic niche provided for a satisfactory estimate of the trophic niche in our study population.

Using carbon and nitrogen stable isotope analysis of blood samples from wild arctic foxes, we tested three hypotheses. *i*) During the pup rearing period, there is a temporal decrease in isotopic niche width of foxes at the population scale due to decreasing accessibility to marine resources. We predicted population isotopic niche to be largest early in the season, when foxes can easily travel on sea ice and thus access both marine and terrestrial habitats. *ii*) Because foxes reproduce in dens where both parents rear the pups, we expected that breeding status and sex of individuals would affect their resource use. We predicted that territorial breeding foxes would have more terrestrial isotopic signatures than potentially more mobile, non-breeding individuals. We also expected that females would be less mobile than males, especially early in the season during lactation, and would thus have more terrestrial isotopic signatures. *iii*) Annual fluctuations in abundance of the main terrestrial prey (cyclic lemmings) should modulate the input from the marine ecosystem and therefore have an impact on individual isotopic ratios. We predicted that terrestrial and marine food webs would be largely decoupled during peak lemming years, due to high availability of the preferred terrestrial prey.

## Materials and Methods

### Ethics Statement

Fox capture and immobilization procedures were approved by the *Université du Québec à Rimouski* Animal Care Committee (permit #CPA32-08-62) and field research was approved by the Joint Park Management Committee of Sirmilik National Park of Canada (permit #SNP-2007–1070).

### Study System

**Figure 1:**
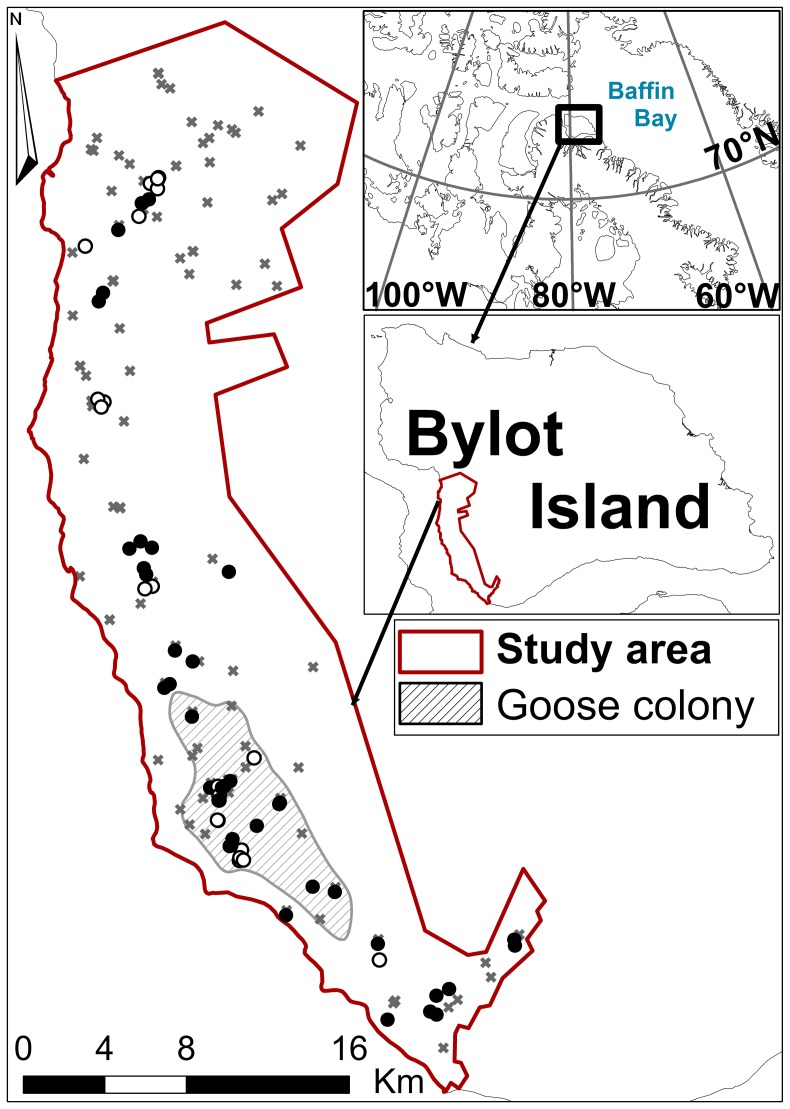
Map of study area showing locations of capture sites relative to the goose colony and fox dens. Locations of capture sites for breeding (•) and non-breeding (○) arctic foxes, monitored denning sites (**X**), and estimated average extent of the goose nesting colony during our study from 2003 to 2008 on Bylot Island (73°N, 80°W), Nunavut, Canada.

We studied arctic foxes on the south plain of Bylot Island (73° N, 80° W) in Sirmilik National Park, Nunavut, Canada. The south plain covers approximately 15% (1,600 km^2^) of the total area of the island and is constituted of a mesic plateau bisected by numerous small streams, which offers suitable denning habitat for arctic foxes [35], [36], [37]. The plain is bordered at the north by high mountains and glaciers. The size of our study area increased from 425 km^2^ in 2003–2005 to 520 km^2^ in 2006–2008 (Fig. 1), thanks to an increased monitoring effort since 2006.

There are two species of lemmings, brown (*Lemmus trimucronatus,* Richardson 1825) and collared (*Dicrostonyx groenladicus*, Traill 1823), and both follow a 3 to 4-year cycle in abundance [38], [39]. Snow depth and duration vary from year to year on Bylot Island [39] but by the end of June it has usually melted in our research area, except for very local patches of deep snow facing northward (Gilles Gauthier, unpublished data). Snow does not prevent predation of lemmings by arctic foxes but may interfere when very deep [39]. Bylot Island also supports a large colony of migratory greater snow goose (*Chen caerulescens atlantica*, Linnaeus 1758) with more than 25,000 pairs breeding during the summer, mostly concentrated in an area of ∼45 km^2^ (Fig. 1; [40]). Foxes prey on lemmings and migratory birds, primarily snow geese [41]. Being opportunistic [21], [42], arctic foxes can also prey on other species, e.g. shorebirds or passerines. However, these species are at low densities on Bylot (e.g. 4±1.3 nests/km^2^ for shorebirds; [43]) and we thus excluded them from our analyses. Inuit hunters from the local community indicate that in winter foxes also feed on marine resources, mainly ringed seal (*Pusa hispida*, Schreber 1775), and that juveniles from this species can be taken by foxes in March-April [44]. They also indicate that seals are heavily hunted by Inuit in the nearby areas until the sea ice melts in July [44], which could potentially lead to the presence of marine mammal carcasses until July along the shore lines. For foxes on Bylot, whelping generally occurs in April-May, lactation lasts until early to mid-July, and pups become independent between mid and late-August (Dominique Berteaux, unpublished data).

### Sampling Design

**Figure 2:**
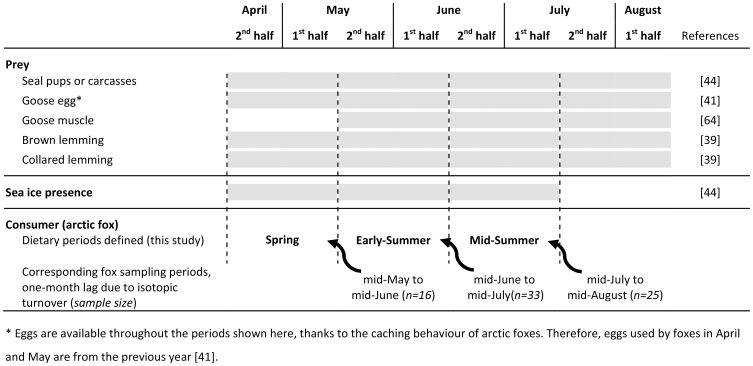
Temporal availability of various food sources used by arctic foxes in the study area on Bylot Island, Canada. We used this phenological information to determine which prey was included in the mixing models for each fox dietary periods (Spring, Early-, and Mid-Summer). We assumed that δ^13^C and δ^15^N in whole blood represented the average diet during the previous month, hence the 1-month lag between dietary periods and their corresponding fox sampling periods (see methods and Fig. S1). Shaded areas are periods of availability (prey) or presence (sea ice).

From 2003 to 2008, we collected 74 whole-blood samples on 60 adult arctic foxes (details in Table 1 and Fig. 2). To cover most of the pup rearing period, we captured arctic foxes continuously from 15 May (earliest) to 18 August (latest) each year all over our study area (Fig. 1). Captures of adults were never attempted directly on den sites. We used padded foot-hold traps (Softcatch #1, Oneida Victor Ltd., USA) and commercial lures to attract foxes. When captured animals were too aggressive to ensure safe handling (about one third of the total number of captures), we anaesthetized them using a combination of medetomidine (0.05 ml/kg) and ketamine (0.03 ml/kg). Before 2007, a mix of xylazine (0.1 ml/kg) and ketamine (0.05 ml/kg) was used instead, but it is unlikely that this change could have interfered with our analyses due to the low concentrations used (<0.01% of total body mass). We then collected 1 ml of blood from the cephalic vein and kept samples in 70% ethanol. We marked individuals with coloured and numbered plastic ear tags (Rototag, Dalton Inc., Ireland). In 2008, we used atipemazole (0.05 ml/kg) as antidote before releasing individuals at their capture site. To confirm breeding status of individuals, we conducted visual observations over a minimum of 12 h at each den showing signs of activity and also at nearby capture sites. Starting in 2006, we used automatic cameras (Reconyx Inc., USA) at dens to confirm reproductive activity of individuals when direct visual observations were not possible (e.g. when we lacked time to observe all dens in a given area). This allowed us to assign breeding status to the majority of the individuals (Table 1) from 2006–2008. From 2003–2005, three individuals were not attributed any breeding status due to lack of information. We did not estimate the age of captured individuals and could therefore not test any age-related hypothesis.

**Table 1:** Number, reproductive status, and sex of arctic foxes sampled annually.

	2003	2004	2005	2006	2007	2008	TOTAL
LEMMING INDEX (n/100 trap nights)	0.0	0.7	0.4	0.2	0.8	0.5	
**NUMBER OF FOXES SAMPLED**							
*Non-breeding*							
Female	0	0	2	5	1	1	9
Male	0	0	1	11	2	1	15
*Breeding*							
Female	1	2	2	2	10	7	24
Male	0	4	0	2	9	7	22
*Undetermined*							
Female	0	0	0	0	0	0	0
Male	0	1	2	0	0	1	4
**TOTAL**	1	7	7	20	22	17	74

The annual index of lemming relative abundance is also indicated. Some foxes were captured more than once (up to three times), hence the total number of individuals is 60 (see methods for details), for a total of 74 samples. Four samples (males of undetermined reproductive status) were not used in the analyses.

We collected prey samples (muscle) opportunistically from fresh carcasses found at fox dens (lemming, goose, seal), using concurrent studies (lemming), or from local hunters (seal). We sampled goose eggs when found freshly predated in their nest or at fox dens. Sample sizes for all prey are reported in Table S1.

### Isotopic Analysis

We froze samples at −80°C (>24 h) and freeze-dried (prey), or oven-dried them at 60°C for >48 h (fox blood), and finally powdered them using mortar and pestle. To avoid any potential bias, we extracted lipids from prey tissues before analysis [45]. Lipid extraction (LE) was done through successive rinsing of powdered samples with 2∶1 chloroform-methanol as a solvent, following the modified method of Bligh and Dyer [46]. LE was done at the Stable Isotopes in Nature Laboratory (SINLab), New Brunswick, Canada. We also tested the effects of LE on ten samples of arctic fox blood; because we detected no effect on carbon or nitrogen isotopic ratios, the remaining samples were not treated. All samples were analysed at the SINLab through combustion in a Carlo Erba NC2500 elemental analyzer before delivery to a Finnigan Mat Delta Plus mass spectrometer (Thermo Finnigan, Bremen, Germany). We express stable isotope ratios of carbon (δ^13^C) and nitrogen (δ^15^N) as ‰ of the deviation from isotopic ratios of international standards, i.e., Pee Dee Belemnite carbonate (PDB) for carbon and atmospheric air (AIR) for nitrogen.


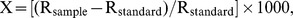


where X (in ‰) is δ^13^C or δ^15^N and R is the absolute isotopic ratio, ^13^C/^12^C or ^15^N/^14^N respectively. Analytical error is reported by providing measures of precision and accuracy [47]. We evaluated overall measurement precision by randomly duplicating a subset of samples; this includes error of precision from the spectrometer and within-sample variation due to lack of homogeneity of powdered samples. Average absolute difference between duplicates was 0.16‰ ±0.05 (95% confidence interval, n = 40) for both δ^13^C and δ^15^N. We estimated accuracy through measurements of isotopic ratios (mean ‰ ±SD) for a commercially available standard (acetanilide, Elemental Microanalysis Ltd.): δ^13^C = −33.6‰ ±0.1 and δ^15^N = −3.2‰ ±0.3 (n = 53).

### Diet Reconstruction Based on Isotopic Mixing Models

We used Stable Isotope Analysis in R (SIAR; [48]) to reconstruct the diet of arctic foxes at the individual and population levels. Our aim was not to investigate the diet in details, but rather to characterize the relative importance of marine *vs.* terrestrial resources during three successive periods of one month each: mid-April to mid-May (thereafter Spring), mid-May to mid-June (Early-Summer), and mid-June to mid-July (Mid-Summer). We defined these 1-month periods *a priori*, based on the availability and phenology of habitat (sea ice) and potential prey species (Fig. 2). These three periods cover most of the pup rearing period of arctic foxes [49]. They also correspond rather well to the periods of availability of the main alternative prey, greater snow goose. Geese typically arrive on Bylot Island at the end of May or early June, and start to nest soon after [50]. They are therefore available only during the second and third dietary periods considered in this study (Early-Summer and Mid-Summer).

Because isotopic turnover in living tissues is not instantaneous, the isotopic ratios of a given tissue sample reflects the ratios of food consumed prior to tissue sampling [51], [52]. Ideally, one would define a unique dietary period for each individual captured. In practice, however, this is rarely possible because prey isotopic signatures are available only sporadically. We therefore pooled captured foxes within 1-month periods. Based on an experimental study on captive arctic foxes [53], we assumed that isotopic ratios of carbon and nitrogen measured in whole blood samples obtained within a given monthly capture period (e.g. from mid-July to mid-August) represented the average diet of the corresponding foxes during the previous monthly period (e.g. from mid-June to mid-July; see Fig. 2 and Fig. S1 for additional details). Thus, for each dietary period, we used the isotopic ratios of consumers that were captured during the following month (Fig. 2).

**Figure 3:**
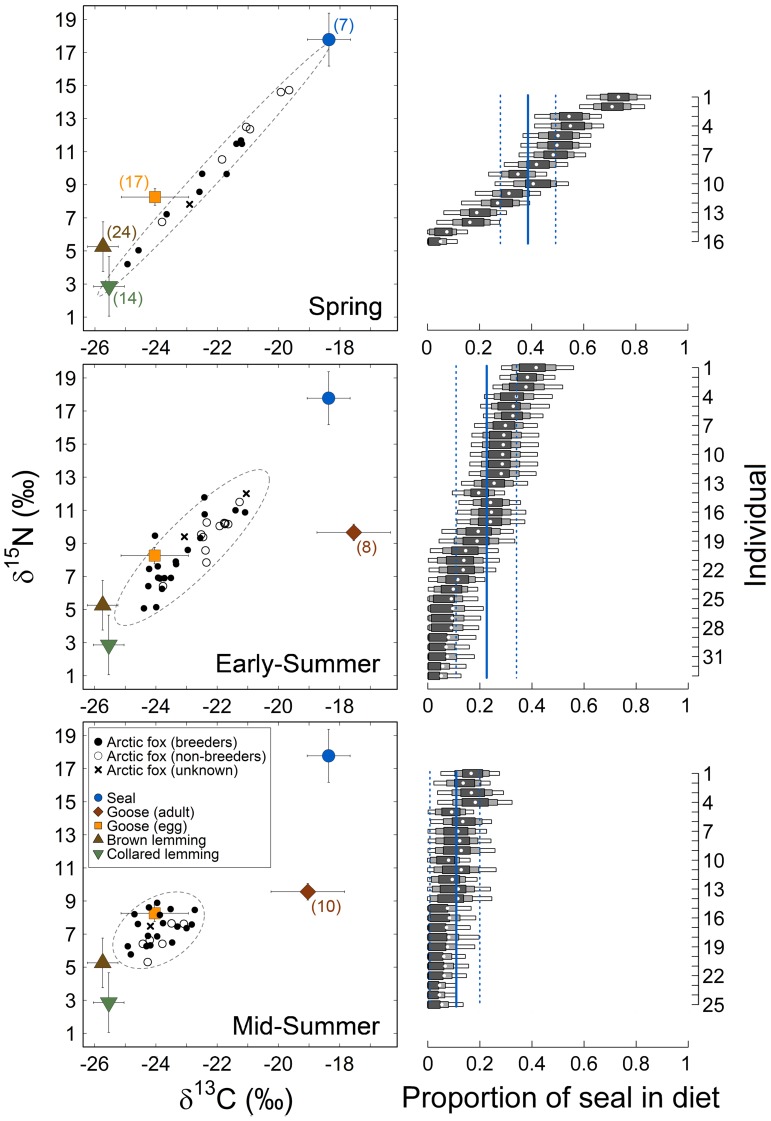
Temporal variation of the isotopic system and the corresponding relative contribution of marine resources to individual arctic fox diets on Bylot Island, Canada. *Left panel* – Isotopic biplots of the isotopic signature (δ^13^C, δ^15^N) of arctic foxes and their potential prey sampled between 2003 and 2008. Dashed grey lines show the 95% CI dispersion ellipses based on standard deviation of foxes’ isotopic ratios for each period of the pup rearing season. Prey sample sizes are indicated in parentheses, unless identical to the previous period (see also Table S1). Spring: diet from mid-April to mid-May; Early-Summer: mid-May to mid-June; Mid-Summer: mid-June to mid-July (Fig. 2). *Right panel* – Corresponding SIAR output distributions of the relative proportion of marine sources (seal) in the reconstructed diet of each individual and by period. We show the mean (white dot) as well as the 50, 75, and 95% Credible Intervals (dark gray, light gray, and white boxes, respectively) of the SIAR posterior probability distributions. For each period, continuous and dotted lines (in blue) show the mean and 95% Credible Intervals at the population level, respectively.

We plotted isotopic ratios of consumers and prey on C–N isotopic biplots for each dietary period separately, taking prey-specific discrimination factors into account [53]. Using the proper discrimination factor (i.e., obtained from the same species in controlled environment) is critical with mixing models, because they are very sensitive to small variations in this parameter [54]. Discrimination factors (Δ^13^C and Δ^15^N) used for arctic fox blood were based on values available from a study on farmed arctic foxes in Ås, Norway [53], and were different for sources from terrestrial or marine origin (Table S1). Due to low and unbalanced sample size among years, with at least two years with no data for each species (Table S1), we could not test adequately for differences in prey isotopic ratios among years and we assumed that prey isotopic ratios did not vary from year to year. Carbon isotopic ratios of goose muscle drifted strongly within the season (i.e., from June to July; −2.8‰, F-ratio test with *df = *1,16, F = 19.78, p<0.001) because, unlike other prey, geese switch from a diet dominated by agricultural food in the south during spring to natural grasses and sedges once in the Arctic (Gauthier et al. 2003). Therefore, we used period-specific isotopic ratios for this source (Fig. 3).

In a given isotopic system (here, C–N isotopic biplot), trophic diversity among individual consumers corresponds to their degree of spread in the isotopic biplot, assuming that all prey have distinct isotopic ratios. This degree of spread can be assessed by measuring the mean distance to centroid: if we consider a group of points in an isotopic bi-plot, and the centroid of all these points, then the average distance to the centroid is the mean distance between each point and that centroid [29], [30]. Therefore, we calculated the centroids of the δ^13^C and δ^15^N values of arctic foxes for each period. In our case, the prey had very distinct isotopic ratios, but the prey isotopic system slightly changed over time (Fig. S2). Therefore, changes in mean distance to centroid in consumer (fox) ratios should be interpreted cautiously.

We ran *siar* mixing models using the following parameters: *iterations  = *3,500,000, *burnin  = *500,000, *thinby  = *10, and flat priors (see [48] for details). *Siar* can account for concentration-dependence in carbon and nitrogen, so we integrated this information in the models.

### Modeling Individual δ^15^N

To examine factors affecting variations in the isotopic niche, we used only δ^15^N as response variable in linear mixed-effects models (LMM) for three reasons. First, carbon and nitrogen isotope ratios of consumers are strongly correlated in this trophic system (Fig. 3 left panel; Pearson’s r = 0.92, p<0.001, *n = *60 individuals). Modeling both δ^13^C and δ^15^N would thus have been redundant, as shown by similar model ranking when using either δ^13^C or δ^15^N as response variables (Table S2 *vs.* S3). Second, the seasonal drift in the C isotopic ratios of goose muscle (X-axis on Fig. 3 left panel) added some uncertainty and made it more difficult to properly discriminate marine from terrestrial sources in Early- and Mid-Summer. Finally, the range of values was higher for δ^15^N than δ^13^C, yielding a better individual discrimination on this axis (Table 2, Fig. 3). For descriptive purposes, one can consider that on the δ^15^N-axis, values >10‰ indicate a predominantly marine signature, while values <9‰ indicate a predominantly terrestrial signature. Note that this approach would not be valid on the δ^13^C-axis due to the overlap between adult goose and seal values. Therefore results from the model selection for δ^13^C as a response variable (Table S3) are not discussed further.

**Table 2:** Sample size, mean, 95% CI, and range of δ^13^C and δ^15^N (‰) values of all fox samples pooled by period.

		δ^13^C‰			δ^15^N‰			Distance to centroid	
	n	mean	95% CI	range	mean	95% CI	range	mean	95% CI
**Spring**	16	−22.1	[−22.9;−21.4]	[−24.9; −19.7]	9.9	[8.4;11.4]	[4.2;14.7]	2.8	[1.7; 3.9]
**Early-Summer**	33	−22.8	[−23.2;−22.5]	[−24.4; −21.1]	8.7	[8.1;9.4]	[5.1;12.1]	1.9	[1.5; 2.3]
**Mid-Summer**	25	−23.9	[−24.2;−23.7]	[−24.9; −22.7]	7.2	[6.9;7.6]	[5.3;8.9]	1.0	[0.8; 1.2]

The mean distance to the centroid of the fox data is also indicated for each period (see details in methods).

We selected models in R.2.12 [55] using the package *lme4* [56]. We used five explanatory variables as fixed effects in LMMs:

- Breeding status (*Breeding*; 2 levels: yes, no). Individuals that were observed providing care to pups at dens were considered breeders. Four individuals whose breeding status could not be confirmed were excluded from the models.
- Time period (*Period*; 3 levels: Spring, Early-Summer and Mid-Summer, Fig. 2). Because the periods of prey availability are rather well defined, with some prey suddenly appearing in the system (e.g. migrating geese), we did not consider models with date as a continuous variable.
- Sex of individuals (*Sex*; 2 levels: male, female).
- Distance to the edge of the goose colony (*Goose*; 2 levels: close, far) was classified as a binary variable. We expected a threshold rather than a linear effect of *Goose* on the δ^15^N of arctic foxes, because this food source (especially goose eggs) becomes marginal beyond a certain distance [42]. We used packages *tree* and *mgcv* [57] to fit a regression tree using δ^15^N as response variable and the smoothed distance to the goose colony as the explanatory variable. The first node of the regression tree split the data into two groups maximizing inter-group variance while minimizing intra-group variance. Based on this, the distance threshold was set at 6.2 km from edge of the colony. The colony contour was delimited in 2007 and 2008, so we used the average extent of the colony during these two years, assuming that the extent of the colony remained similar throughout the study period.
- Index of annual lemming abundance as a continuous variable (*Lemming*; number captured/100 trap nights). An index of lemming abundance was estimated yearly for both species simultaneously through snap-trapping at two different sites situated ∼30 km apart within our study area. It represented a mean of 1826±311 SD trap nights per year [38], [58]. Lemming trapping is part of the long term monitoring on Bylot Island and details of sampling design can be obtained in Gruyer et al. [36].

**Figure 4:**
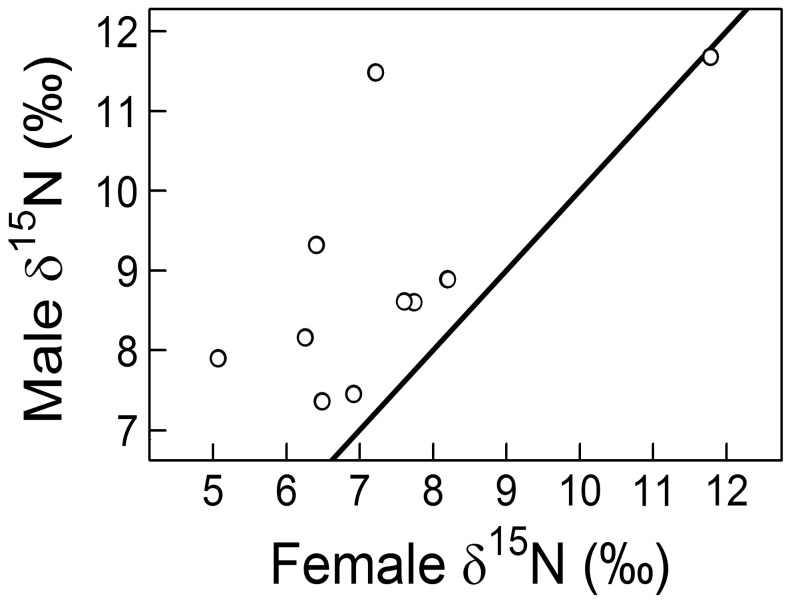
Comparison of δ^15^N (‰) within arctic fox breeding pairs. Dots above the line show pairs where the male had a higher δ^15^N than the female. Pearson’s r = 0.67, t = 2.57, *df = *8, p = 0.033.

Forty-nine individual foxes were captured once, eight were captured twice, and three were captured thrice. Moreover, 10 breeding pairs were captured. Paired individuals could have caused pseudo replication in our data because the δ^15^N signature was correlated between paired males and females (Pearson’s r = 0.67, t = 2.5651, *df = *8, p = 0.033; Fig. 4). Thus, we used both identity (*Fox ID*) and breeding pair (*Pair*) as non-nested random factors in our LMM.

We constructed twelve models *a priori*, corresponding to plausible hypotheses (*sensu* [59]). For instance, due to sexual differences in parental care [49], we tested interactions between *Sex* and *Breeding*, expecting that breeding females would have more terrestrial isotopic ratios (lower δ^15^N). We also predicted that differences in δ^15^N due to breeding status would vary with the period considered and thus tested for potential interactions between *Period* and *Breeding*.

Sample sizes were too small to consider models with >2 interactions. We used the maximum likelihood (ML) method during the selection process and the Akaike Information Criterion corrected for small sample size (AIC_c_) to determine which model was better supported by our data [59]. However, we estimated model parameters using the restricted maximum likelihood (REML) method, which generates more conservative estimates of variance components ([60], p. 150–151). We calculated credible intervals (95% CI) for all estimates using Markov Chain Monte Carlo methods (MCMC) resampling from the posterior distributions of the parameters of the top-ranking model (10^6^ iterations).

Following modeling of δ^15^N, we wished to describe the relationship between the proportion of breeding foxes and the abundance of lemmings. We used a generalized linear mixed-effects model (GLMM) with a binomial error distribution and a logit link function using the R package *lme4* [56], with the breeding status of captured foxes as a binomial response variable. We used our global index of lemming abundance as the main fixed effect, and the identity of foxes (*Fox ID*) as a random effect. We included only year 2004 to 2008, because sample size was too low in 2003 to estimate the proportion of breeders. We evaluated the significance of the main effect by calculating the change in deviance from a null model (i.e., intercept only) and comparing it to the χ^2^ distribution for the GLMM.

## Results

### Population Isotopic Niche and Individual Variation in Resource Use

The results support our first hypothesis of a general decrease in isotopic niche width from Spring to Mid-Summer. Ranges in carbon and nitrogen isotopic ratios decreased more than two-fold between Spring and Mid-Summer (Table 2 and Fig. 3 left panel). In addition, average population δ^13^C and δ^15^N were, based on 95% CI, significantly lower in Mid-Summer than in the two previous periods (Table 2). As predicted, both the shift and reduction in the δ^15^N range denoted a change in diet through time, from a mix of marine and terrestrial in Spring to almost exclusively terrestrial in Mid-Summer (Fig. 3, right panel).

We found high inter-individual variation in Spring; five individuals fed mostly on marine sources, whereas nine fed mostly on terrestrial sources (i.e., all sources but seal), particularly lemmings, and the rest adopted a balanced diet between marine and terrestrial resources (Fig. 3, right panel). Intra-population variation in diet (average distance to the centroid) decreased significantly from Spring (2.8±1.0‰, 95% CI) to Mid-Summer (1.0±0.2‰, 95% CI; Table 2). Higher isotopic niche width in Spring was thus associated with higher inter-individual variation in the use of marine *vs.* terrestrial food sources. The reconstructed individual diets illustrate inter-individual differences in use of marine sources (seal) for each period (Fig. 3 right panel). Estimated average relative contribution of marine sources at the population level decreased from 0.39 (95% CI =  [0.28;0.49]) in Spring to 0.23 [0.11;0.34] in Early-Summer and 0.11 [0.00;0.20] in Mid-Summer, when the 95% CI included zero. Hence, we confirmed that the decrease in isotopic range and average distance to centroid was indeed due to a shift toward a more uniformly terrestrial diet for the population, corresponding to lower δ^15^N values.

### Effects of Individual Characteristics on Resource Use

**Figure 5:**
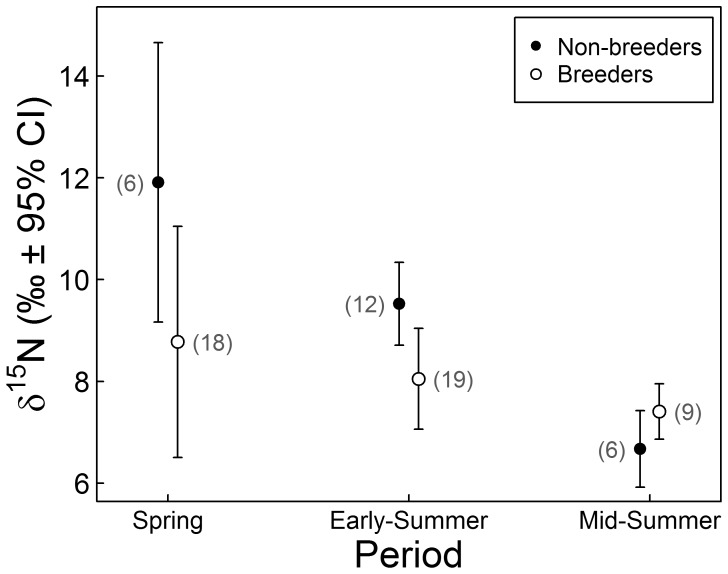
Seasonal variation in δ^15^N (mean ‰ ±95% CI) of male and female arctic foxes. Average δ^15^N (‰ ±95% CI) of arctic foxes on Bylot Island, Nunavut, based on their breeding status and period of the pup rearing season. Spring: mid-April to mid-May; Early-Summer: mid-May to mid-June; Mid-Summer: mid-June to mid-July (Fig. 2). Numbers in parentheses indicate sample sizes and all data from years 2003–2008 were pooled, except those from four individuals whose breeding status could not be determined (Table 1).

We also found support for our second hypothesis: according to the most supported model (Table S2), non-breeding foxes had higher δ^15^N in Spring (+5.0‰ on average; Table 3), but this gap lessened in Early- and Mid-Summer (interaction between *Period* and *Breeding*). This model confirmed a strong temporal trend in δ^15^N, with average δ^15^N being smaller in Mid-Summer than in Spring (−1.1‰ on average; Table 3). Therefore, individual differences in nitrogen isotopic ratios due to breeding status dampened over the course of the summer, through a sharp decrease of δ^15^N in non-breeders and a slight decrease in breeders (Fig. 5). The distance from the goose colony had an influence on individual δ^15^N, which confirmed the existence of a spatial pattern in isotopic ratios. Individuals captured far from the goose colony (i.e., ≥6.2 km) had smaller δ^15^N (difference of −2.8±0.8‰, 95% C.I) than those captured closer. Males had higher δ^15^N on average than females (difference of +1.7±0.8‰, 95% CI), but only if they were breeding. In all but one of the ten captured breeding pairs, the male had a higher δ^15^N than the female (Fig. 4). In non-breeders, δ^15^N was similar for both sexes when accounting for a compensating interaction between *Sex* and *Breeding*.

**Table 3:** Estimated parameters for the most parsimonious model selected.

Random effects	Standard deviation	
Fox ID (intercept)	0.8	
Pair (intercept)	0.5	
Residual	0.8	
**Fixed effects**	**Estimates**	**95% CI**
Intercept	8.1	[7.3;9.3]
* Breeding* (No)	5.0	[3.2;6.2]
* Sex* (Male)	1.7	[0.9;2.4]
* Period* (Early-Summer)	0.1	[−1.3;0.8]
* Period* (Mid-Summer)	−1.1	[−2.5; −0.4]
* Goose* (Far)	−2.8	[−3.6; −2.0]
* Breeding* (No) * *Sex* (Male)	−2.0	[−3.3; −0.6]
* Breeding* (No) * *Period* (Early-Summer)	2.8	[−4.2; −0.8]
* Breeding* (No) * *Period* (Mid-Summer)	−2.6	[−4.3; −0.6]

Parameter values (‰ δ^15^N, with 95% Credible Interval) were estimated for the general linear mixed-effects model that received the best support among all candidate models (Table S2). The fixed intercept represents the estimated average δ^15^N in spring (*Period*), for females (*Sex*) that were breeding (*Breeding*) close to the goose colony (*Goose*).

### Lemming Cycles and Resource Use at the Individual Level

**Figure 6:**
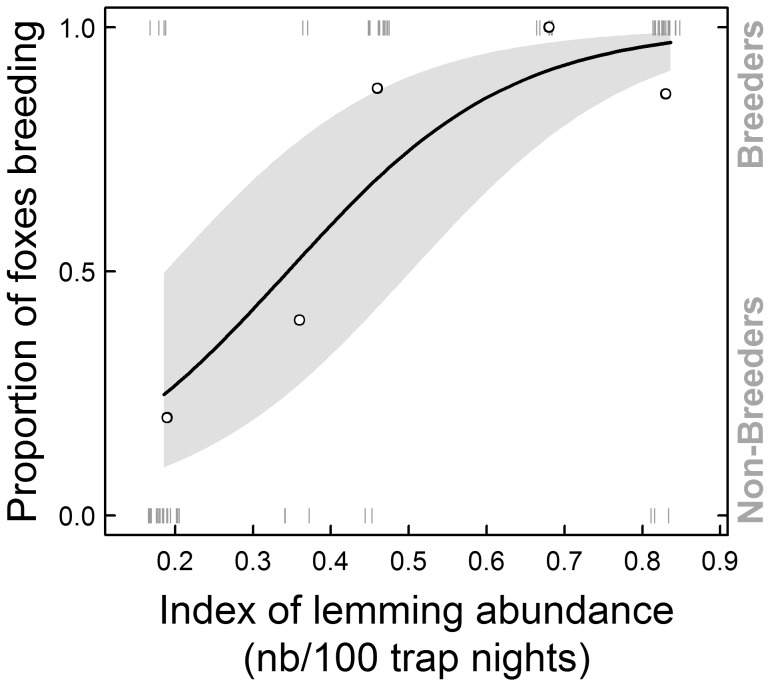
Proportion of breeding foxes *vs.* lemming trapping index during the study period. Proportion of breeding foxes captured annually as a function of the lemming snap-trapping index. The curve represents predictions from a generalized linear mixed-effects model fitted to the data (±1SE, shaded area around the curve). Small vertical bars (gray) represent the original data for breeders (top) or non-breeders (bottom) to which the model was fitted. The bars were jittered (randomly displaced over small distances on the X-axis) in order to better show data concentration. The open circles show the actual proportion of breeders for a given year/lemming index value. Total number of foxes captured each year is also available in Table 1. Data include only year 2004 to 2008 and lemming abundance data from 2004 to 2006 are drawn from Morrissette et al. [58], based on our long term monitoring of lemming abundance on Bylot Island (see methods for details).

We could not find support for our third hypothesis that lemming cycles had a direct influence on individual δ^15^N. *Lemming* was excluded from the top model but was retained in our second most parsimonious model, which received less support (ΔAIC_C  = _3.0; Table S2) and differed from the best model by only one parameter. The model including *Lemming* had a similar log-likelihood value and the confidence interval for the parameter estimate of *Lemming* [−1.1, +1.7‰] overlapped zero. Therefore, we concluded that *Lemming* did not explain variation in individual δ^15^N. Nonetheless, we checked for any potential indirect influence of lemming cycles on the diet of the whole population by evaluating the relationship between *Lemming* and the proportion of breeders among the foxes captured annually. There was a significant effect of *Lemming* on the proportion of breeders when compared to a null (intercept only) model (χ^2^ = 24.795, *df = *1, p<0.0001) (Fig. 6). Thus, a higher annual proportion of breeders was indeed associated to higher lemming abundance. We address this issue further in the discussion.

## Discussion

In an opportunistic and long-ranging carnivore dwelling both the arctic tundra and the sea ice, we expected that high behavioural flexibility among individuals would generate intra-population variation in the feeding niche. Spatial variation in resource use was documented earlier between arctic fox populations at large scale [61]. At a finer scale, it was suggested that within-population differences in diet could be associated with variation in resource distribution [44], [61], [62]. This is also supported by a recent study showing an effect of access to marine resources on the trophic niche breadth of arctic foxes at regional spatial scales [15]. Our study addresses the causes of diet variation among individuals at fine spatial (km) and temporal (month) scales and suggests that individual characteristics, such as breeding status and sex, can also generate intra-population variation in resource use.

Because our results occasionally depict small differences in individual isotopic ratios, it is important to consider what differences are biologically meaningful. Proper interpretation of differences in isotopic ratios should account for basal inter-individual variation between individuals fed on an isotopically homogenous diet [63]. Data from a study on farmed arctic foxes [53] reported standard deviations of ±0.2‰ for δ^13^C and ±0.4‰ for δ^15^N for blood cells signatures of 17 foxes fed the same diet. Both values are well below the inter-individual differences observed here, indicating that this variation cannot be solely explained by factors such as nutritional stress or age. Finally, there is no evidence that age or experience affect foraging capacity and body condition of arctic foxes [62], so even though we could not determine the age of individual foxes sampled, resource use and δ^15^N values should not be affected by this variable.

### Population Isotopic Niche and Individual Variation in Resource Use

Individual variation and specialization in resource use can change rapidly, e.g. on a seasonal basis [11]. For instance, a population formerly composed of generalists and specialists could become homogenously generalist in response to change in prey availability. The isotopic niche of our arctic fox population decreased rapidly throughout the pup rearing period, mainly due to a strong decrease in use of marine resources. This rapid decoupling of the terrestrial and marine food chains could be related with three events linked to prey accessibility and availability. First, an increase in accessibility of lemmings with the disappearance of snow. Second, an increase in availability of terrestrial prey, particularly snow geese arriving in large numbers in late May to nest in the area [64]. Third, a decrease in the accessibility of marine prey, in particular seals, which can constitute an important food source for arctic foxes in winter and spring [22]. Growing seal pups become more difficult to catch as spring progresses [65]. The melting of the landfast ice surrounding Bylot Island, usually completed by late July but starting more than one month earlier, can also reduce accessibility to marine prey in summer, although carcasses washing ashore or left by local hunters can still be used [44].

Early in the season, there was a strong alignment of resource use along a continuous lemming-seal gradient, including apparent lemming- or seal-specialists. We did not find a bi-modal distribution of isotopic ratios in Spring, but rather a continuum from mainly terrestrial to mainly marine diets indicating that arctic foxes foraged predominantly either on sea ice, on land, or both, confirming results from other studies [15], [22]. Based on the isotopic biplots, there were apparently no foxes foraging exclusively on lemmings later in Early- or Mid-Summer, when lemming density and accessibility (absence of snow cover) is highest [39]. Although other species such as migratory birds are present only during that period, we expected arctic foxes to preferentially select small rodents when available. Our results suggest that arctic foxes are not necessarily opportunistic specialists on Bylot Island [21]. They rather seem to be versatile and generalist foragers. This characteristic, associated with their high mobility [24], allows them to temporarily link the food chains from two distinct ecosystems: the arctic tundra and the nearby marine environment. There is no evidence that high individual variation could be due to differential accessibility of marine resources, especially in spring when the sea ice is present. Our study area includes a large proportion of shoreline and most capture sites were situated within a few kilometres of the shore. Arctic foxes can cover large distances at high speeds (up to 90 km/day [24]) and could easily access marine habitat within a few hours. Thus, we assumed that captured foxes all had access to seals reproducing on the nearby landfast ice in spring [44]. Later in summer however, an increase in territoriality (and the inherent need to defend a territory), in addition to the need to stay close to the den to feed pups, could prevent some foxes to cover larger distances to forage.

### Causes of Individual Variation in Resource Use

Individual variation in resource use was clearly linked to breeding status, spatial distribution of resources (in relation to the goose colony), and sex. Non-breeders foraged more on marine resources than breeders in spring, but not later in summer when individuals used almost exclusively terrestrial resources, regardless of breeding status. The exact mechanism through which resource partitioning occurred between breeders and non-breeders in spring is still unknown, but intra-specific competition and territoriality could offer some explanation. Darimont *et al.* [10] suggested that greater inter-individual variation was due to increased intra-specific competition in grey wolves. In Iceland, coastal populations of arctic foxes had higher inter-individual variation in isotopic ratios than their inland counterparts [61]. It was also suggested that this was a consequence of territoriality and differential access to marine resources, with territories situated near the shoreline providing better access to these resources [15]. Our study area did not feature a sufficiently large shoreline-inland gradient to test this hypothesis. The pattern we observed might be explained by greater mobility of non-breeding individuals, in addition to their exclusion from the inland areas by breeding and territorial individuals early in the season. Further testing of this hypothesis would require tracking of individuals before sampling their tissues for isotopic analysis.

The observed spatial trend in δ^15^N was due to the goose colony, a dense patch of resources covering ∼9% of the study area. Individuals situated far from the colony had lower δ^15^N likely because they consumed more lemmings, a prey with lower nitrogen ratios. This spatial trend has also been found in the diet of fox pups and shows how this resource subsidized by southern ecosystems is diffused through the arctic tundra ecosystems [42]. Our sample size was too small to test how this spatial trend interacted with the seasonal trend discussed above.

We detected inter-sex differences in δ^15^N only for breeding individuals. In controlled conditions, adult male and female arctic foxes have similar isotopic ratios when fed on the same diet [53]. Although we cannot not reject the hypothesis that breeding males tended to forage more on marine resources than breeding females, small inter-sex difference and the generally low δ^15^N values of breeders, suggest an alternative explanation. The observed difference could indeed be due to different metabolic processes, particularly lactation. Few studies have addressed the question of isotopic ratios during lactation in wild mammals (but see [66], [67]), but lactation can induce lower nitrogen isotopic ratios [68]. Our sample size was too small to address this question thoroughly, but these results call for more research in order to elucidate the role of lactation, and more generally of physiological stress, on isotopic ratios in wild mammals.

### Cyclic Lemmings and Population Trophic Niche

There was no effect of inter-annual variation in lemming abundance on the δ^15^N of individual arctic foxes. This result is surprising, given the numerous studies showing the strong influence of fluctuations of rodent abundance on arctic predators [69], [70], [71], [72], including at our study site [73]. One could argue that our index was too coarse to actually track trends in the actual abundance of lemmings. However, this relatively simple estimate explained very well the breeding output of snowy owls (*Bubo scandiacus*, Linnaeus 1758), the predation pressure on foxes’ alternative prey such as geese [58], [64], and the proportion of breeding foxes on our study area (Fig. 6). Moreover, it is important to note that throughout our study period, values for the index of lemming abundance were always <1.0 lemming per 100 trap nights even during peak years, while earlier values (1993–2002; [38]) were sometimes >4.0 during peak years. Other studies on lemmings conducted in the Canadian North have reported snap-trap indices >7.0 lemming 100 trap nights [74], but they were conducted in areas of higher primary productivity such as the western Canadian arctic [75], making direct comparisons difficult. However, one explanation for our results could be that lemming peaks were not pronounced enough to generate detectable effect in the fox diet at the individual level.

In fact, another and more plausible explanation for our results is that lemming cycles have an indirect effect on the use of marine resources at the population level, mediated by a direct effect on the proportion of breeding foxes, as we showed. Therefore, during years of high lemming abundance, non-breeders still feed at least partly on marine resources although they can represent <10% of the total number of adults that we captured/observed on Bylot Island (compared to >60% in a year of low lemming abundance). However, the non-breeder δ^15^N would remain high in all years, independent of lemming abundance. Therefore, lemmings would have a lower indirect effect at the population level on the absolute quantity of marine resources entering the terrestrial system through the foraging of non-breeding foxes.

### Potential Impacts of Allochthonous Inputs on Arctic Tundra Food Webs

Connections between marine and terrestrial food chains can have far-reaching implications, including circulation of pollutants between food webs [76] and changes in terrestrial plant communities [77]. In some tundra ecosystems, input of marine resources helps explaining the persistence of invasive species such as the red fox (*Vulpes vulpes,* Linnaeus 1758), which can rely on these resources when terrestrial prey become too scarce [20]. Many terrestrial carnivores connect both the terrestrial and marine food webs, but when they do, they often capture prey entering or coming very close to their terrestrial habitat such as nesting marine birds, spawning salmon, or invertebrates in littoral areas [11], [78], [79]. The arctic fox is one of the rare carnivores that dwell in both terrestrial and marine habitats for extended periods of time, and as such may act as a strong and dynamic link connecting terrestrial and marine food webs. In doing so, they may further the impact of allochthonous subsidies in the terrestrial ecosystem by increasing predation pressure on their prey, thus leading to top-down control of herbivores [80].

On Bylot Island, there is evidence of top-down control of lemming populations by their predators, and allochthonous subsidies likely strengthen this effect [37], [43]. Other lemming predators, such as snowy owls, have recently been shown to feed extensively in marine habitats in winter [81], which could also amplify their subsequent impact on lemming populations. Investigating the causes of individual variation in use of allochthonous resources by arctic terrestrial predators is a step towards a better quantification of the role of these inputs into the arctic tundra food web. Although in our study population only a minority of foxes were strongly using marine resources in spring, the average proportion of marine resources used at the population level remained globally high in the same period. Knowing that arctic foxes can rely on marine resources in other periods of the year, such as winter [22], [82], a logical next step would be to focus on the potential effects of this behaviour on the population dynamics of this species. Though it remains to be shown at larger temporal scales, a strong reliance of this top predator on the marine ecosystem and sea ice could hinder its capacity to adapt to a changing climate.

## Supporting Information

Figure S1 **Schematic view of the rationale behind the attribution of dietary periods to a given period of fox captures and sampling for isotopic analysis.** The coloured circles represent three hypothetical capture events at the beginning (blue), in the middle (green), and at the end (orange) of one capture period (e.g., in this case August). The coloured bars show the corresponding individual dietary periods covered by the blood samples, which are of about two months each. Isotopic ratios of all individual foxes captured during this given one-month period were pooled together and assumed to be representative of the average diet during the previous month (in this case, July). This assumed dietary period was chosen because it covers 50% of the actual dietary period of any fox that was sampled in the corresponding capture period. Therefore, we assumed that, on average, the previous month represented well the dietary period of foxes captured during a given month.
(TIF)

Figure S2 **Biplots of isotopic ratios for the main potential prey of arctic foxes on Bylot Island, Nunavut.** The isotopic biplots (δ^13^C, δ^15^N) show the centroid of the prey’s cloud of points (cross) and the distance to the centroid for each prey type (dotted lines). Value of the mean distance to centroid for each period is also provided, and represents the average spread of the prey isotopic data in the δ^13^C-δ^15^N biplot. Prey sample sizes are in Table S1 and Fig. 3 left panel.
(TIF)

Table S1 **Sample sizes for prey tissues used in dietary analyses, grouped by year.** The last two columns report the discrimination factors (Δ^13^C and Δ^15^N) used as parameters in isotopic mixing models.
(DOC)

Table S2 **Results of the general mixed-effects model selection using maximum likelihood, for the nitrogen isotopic ratio (δ^15^N ‰) in adult arctic foxes on Bylot Island, Nunavut.** The model with the most support based on the data is shown in bold. *K = * number of parameters*; LogLik  = * Log-likelihood.
(DOC)

Table S3 **Results of the general mixed-effects model selection using maximum likelihood, for the carbon isotopic ratio (δ^13^C ‰) in adult arctic foxes on Bylot Island, Nunavut.** The model with the most support based on the data is shown in bold. *K = * number of parameters*; LogLik  = * Log-likelihood.
(DOC)
