# Supplementary figures and images for: The Marine Side of a Terrestrial Carnivore: Intra-Population Variation in Use of Allochthonous Resources by Arctic Foxes
Source: PLoS One. 2012 Aug 3;7(8):e42427. doi: 10.1371/journal.pone.0042427 (PMC3411752; doi:10.1371/journal.pone.0042427)

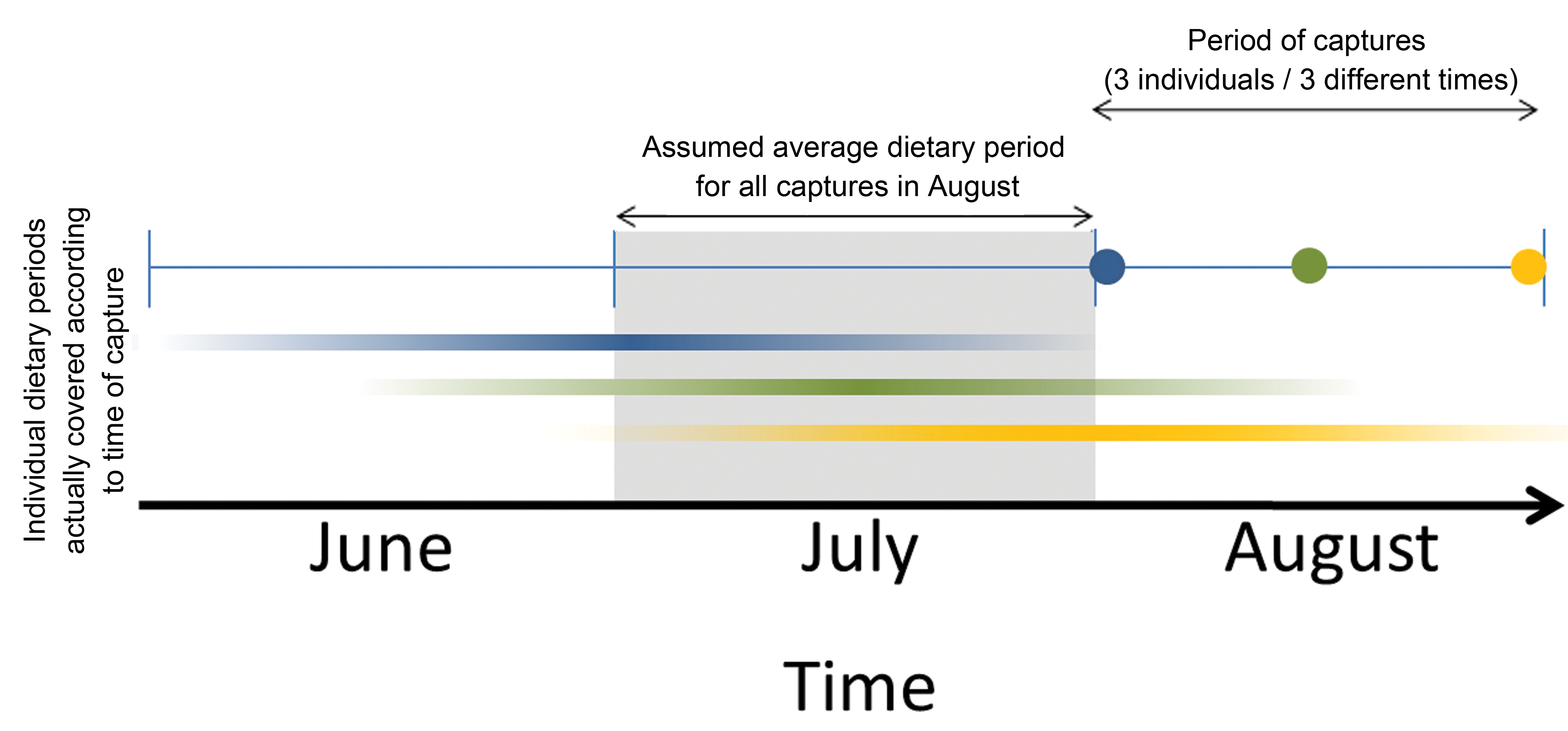

Supplement: Figure S1 — Schematic view of the rationale behind the attribution of dietary periods to a given period of fox captures and sampling for isotopic analysis. The coloured circles represent three hypothetical capture events at the beginning (blue), in the middle (green), and at the end (orange) of one capture period (e.g., in this case August). The coloured bars show the corresponding individual dietary periods covered by the blood samples, which are of about two months each. Isotopic ratios of all individual foxes captured during this given one-month period were pooled together and assumed to be representative of the average diet during the previous month (in this case, July). This assumed dietary period was chosen because it covers 50% of the actual dietary period of any fox that was sampled in the corresponding capture period. Therefore, we assumed that, on average, the previous month represented well the dietary period of foxes captured during a given month. (TIF) [file pone.0042427.s001.tif]

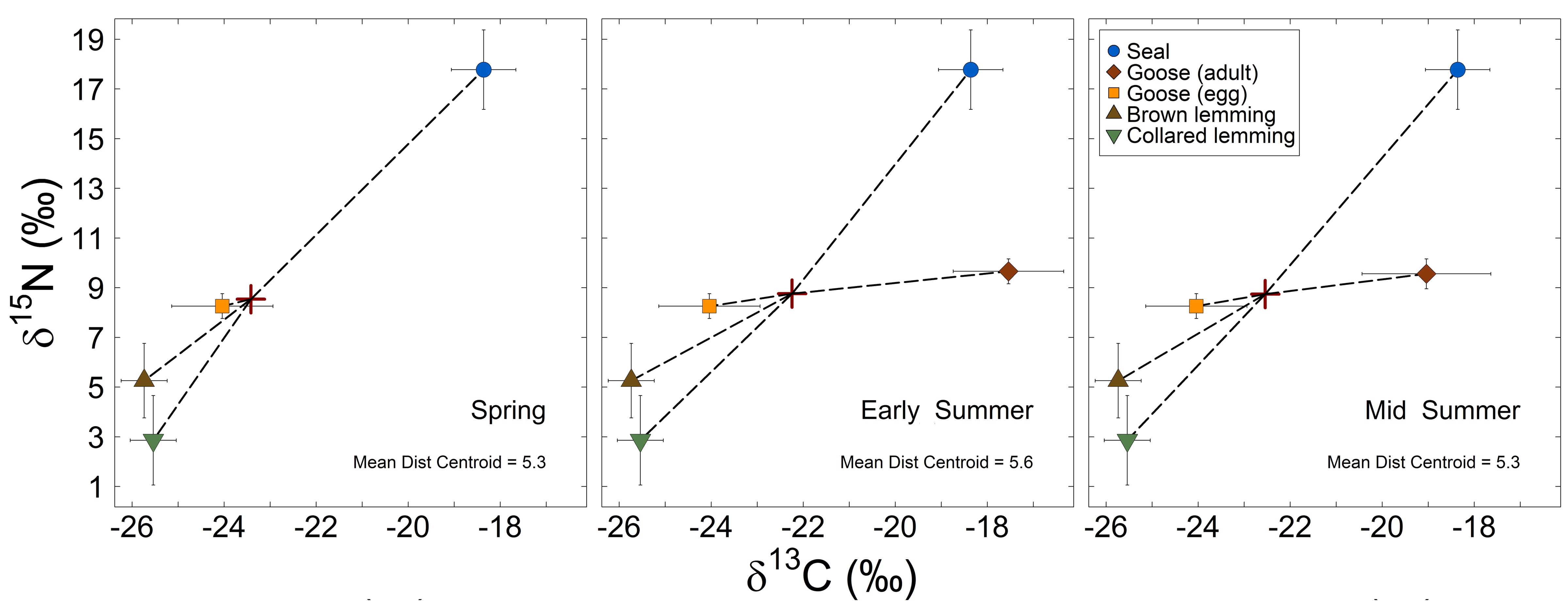

Supplement: Figure S2 — Biplots of isotopic ratios for the main potential prey of arctic foxes on Bylot Island, Nunavut. The isotopic biplots (δ13C, δ15N) show the centroid of the prey’s cloud of points (cross) and the distance to the centroid for each prey type (dotted lines). Value of the mean distance to centroid for each period is also provided, and represents the average spread of the prey isotopic data in the δ13C-δ15N biplot. Prey sample sizes are in Table S1 and Fig. 3 left panel. (TIF) [file pone.0042427.s002.tif]
