# Supplementary material for: The Marine Side of a Terrestrial Carnivore: Intra-Population Variation in Use of Allochthonous Resources by Arctic Foxes
Source: PLoS One. 2012 Aug 3;7(8):e42427. doi: 10.1371/journal.pone.0042427 (PMC3411752; doi:10.1371/journal.pone.0042427)
Supplement: Table S1 — Sample sizes for prey tissues used in dietary analyses, grouped by year. The last two columns report the discrimination factors (Δ13C and Δ15N) used as parameters in isotopic mixing models. (DOC) [file pone.0042427.s003.doc]

|  |  | **Year** | | | | | |  | **Discrimination factor** | | |
| --- | --- | --- | --- | --- | --- | --- | --- | --- | --- | --- | --- |
| **Prey type (*sample size*)** |  | **2003** | **2004** | **2005** | **2006** | **2007** | **2008** |  | **Δ^13^C**  **(‰  ± SD)** | **Δ^15^N**  **(‰  ± SD)** | |
| Seal (*n = 7*) |  | - | - | - | 6 | - | 1 |  | 0.24 ± 0.16 | 0.38 ± 0.16 | |
| Goose egg (*n = 17*) |  | - | 6 | 5 | - | 5 | 1 |  | 0.56 ± 0.12 | | 1.56 ± 0.12 |
| Goose muscle (*n = 18)* |  | - | 3 | 3 | 5 | - | 7 |  | 0.56 ± 0.12 | | 1.56 ± 0.12 |
| Brown lemming (*n = 24*) |  | - | 5 | 4 | 1 | - | 14 |  | 0.56 ± 0.12 | | 1.56 ± 0.12 |
| Collared lemming (*n = 14*) |  | 1 | 4 | 5 | - | - | 4 |  | 0.56 ± 0.12 | | 1.56 ± 0.12 |
