# Supplementary material for: The Marine Side of a Terrestrial Carnivore: Intra-Population Variation in Use of Allochthonous Resources by Arctic Foxes
Source: PLoS One. 2012 Aug 3;7(8):e42427. doi: 10.1371/journal.pone.0042427 (PMC3411752; doi:10.1371/journal.pone.0042427)
Supplement: Table S3 — Results of the general mixed-effects model selection using maximum likelihood, for the carbon isotopic ratio (δ13C ‰) in adult arctic foxes on Bylot Island, Nunavut. The model with the most support based on the data is shown in bold. K = number of parameters; LogLik = Log-likelihood. (DOC) [file pone.0042427.s005.doc]

| Model (random factor: *Fox ID + Pair*) | *k* | LogLik | Δ AIC_C_ | AIC_c_ weight |
| --- | --- | --- | --- | --- |
| ***Breeding* * (*Sex* + *Period*) + *Goose*** | **12** | **-67.92** | **165.32** | **0** |
| *Breeding* * (*Sex* + *Period*) + *Goose* + *Lemming* | 13 | -67.84 | 168.19 | 2.87 |
| *Lemming* * *Period* + *Breeding* * *Sex* + *Goose* | 13 | -68.35 | 169.19 | 3.87 |
| *Period* * (*Breeding* + *Lemming*) + *Goose* + S*e*x | 14 | -69.13 | 173.91 | 8.59 |
| *Lemming* * *Goose* + *Breeding* * *Period* + *Sex* | 13 | -70.85 | 174.21 | 8.89 |
| *Lemming* + *Period* + *Goose* + *Sex* + *Breeding* | 10 | -75.42 | 174.57 | 9.25 |
| *Lemming* * (*Period* + *Goose*) + *Sex* + *Breeding* | 13 | -73.07 | 178.65 | 13.33 |
| *Breeding* * (*Sex* + *Period*) + *Lemming* | 12 | -79.25 | 187.98 | 22.66 |
| *Period* * (*Breeding* + *Lemming*) + *Goose* | 13 | -77.93 | 188.35 | 23.03 |
| *Lemming* * (*Sex* + *Period*) + *Goose* | 12 | -82.14 | 193.76 | 28.44 |
| *Breeding* + *Lemming* | 6 | -104.93 | 223.19 | 57.87 |
| *intercept only* | 4 | -112.78 | 234.17 | 68.85 |

Model parameters: *Breeding* = breeding status (yes or no), *Goose* = shortest distance to the edge of the goose colony (close or far), *Lemming* = Index of lemming density (number/100 trap-nights), *Period =* Time period (Spring, Early-Summer, or Mid-Summer), *Sex* (male or female). See main text and table 2 for details. Identity of foxes (*Fox ID*) and breeding pair (*Pair*) were used as random factors.
